# Associations between dietary intake, physical activity, and obesity among public school teachers in Jeddah, Saudi Arabia

**DOI:** 10.3389/fnut.2023.1081928

**Published:** 2023-01-24

**Authors:** Noha M. Almoraie, Israa M. Shatwan, Maha A. Althaiban, Mahitab A. Hanbazaza, Huda A. Wazzan, Najlaa M. Aljefree

**Affiliations:** Department of Food and Nutrition, Faculty of Human Sciences and Design, King Abdulaziz University, Jeddah, Saudi Arabia

**Keywords:** obesity, dietary behaviour, diet, physical activity, schoolteachers

## Abstract

**Objective:**

We aimed to assess the dietary intake of certain food groups in a representative sample of public-school teachers living in Jeddah city. We also, examined the association of dietary intake with physical activity and obesity among schoolteachers.

**Methods:**

The study was a cross-sectional online survey, conducted among 640 (177 male, 463 female) schoolteachers aged between 20 and 62 years old and working in public primary, intermediate, and high schools in Jeddah. Measurements included gender, weight, height, body mass index (BMI), health problems, and lifestyle behaviours, including physical activity levels, smoking status, and dietary intake.

**Results:**

Based on gender, number of non-smoking women (94%) was higher than number of non-smoking men (57.1%) (*P* < 0.001). However, men were more active than women (*P* = 0.03). Regarding BMI, there were more overweight men than women, while obese women numbered more than men (*P* = 0.003). There was no significant difference in dietary intake between men and women except that men consumed more soft drinks than women (*P* = 0.002). Lower physically active schoolteachers were less likely to consume salad (OR = 0.6, 95% CI 0.4–0.9; *P* = 0.02), vegetables (OR = 0.6, 95% CI 0.3–0.9; *P* = 0.01), beans and legumes (OR = 0.4, 95% CI 0.2–0.7; *P* = 0.005), wholegrain bread (OR = 0.6, 95% CI 0.4–0.9; *P* = 0.03), dairy products (OR = 0.6, 95% CI 0.4–0.9; *P* = 0.01), snacks (OR = 0.5, 95% CI 0.2–0.8; *P* = 0.01), and fish (OR = 0.4, 95% CI 0.2–0.9; *P* = 0.04) compared to those with high levels of physical activity. Only fruit intake was considered statistically significant (OR = 0.4, 95% CI 0.3–0.7; *P* = 0.003). The study found a relationship between the BMI of schoolteachers and food intake. Obese schoolteachers had lower consumption of fruits (OR = 0.3, 95% CI 0.2–0.7; *P* = 0.007) and white meat (OR = 0.5, 95%CI 0.3–0.9; *P* = 0.03) than schoolteachers in the normal weight group.

**Conclusion:**

The high prevalence of physical activity, dietary intake and body weight among Saudi teachers is a major public health concern. The present study identified several lifestyle factors associated with body weight that may represent valid targets for the prevention and management of obesity among Saudi school teachers. Promoting active lifestyles and healthy diets would be primary targets for obesity prevention.

## 1. Introduction

Obesity and overweight are major public health problems that are considered a global pandemic (1) due to disease and biological risk factors linked to non-communicable diseases. The World Health Organization (WHO) defines obesity and overweight as abnormal or excessive accumulation of fat and classifies it according to a body mass index (BMI) of higher than 25 and 30 kg/m^2^, for overweight and obesity, respectively (2). The prevalence of adult obesity has increased threefold globally in the last four decades (3), according to WHO. There is a growth in obesity rates around the world (>1 billion). In Europe, two thirds of the adult's population is obese (3), whereas, in Saudi Arabia, more than half the adults are considered obese, which is also the case in Arabian gulf countries (4). Obesity and overweight causes are complicated by environmental, genetic, behavioural, and socioeconomic factors. It is well-known that dietary intake and physical activity are risk factors linked with obesity and overweight (5). In Saudi Arabia, obesity and overweight increased due to shifting lifestyles as a result of the growth of the economy, technology, food availability, variety, and changes in the population's living standards. This led to changes in food choice, energy intake and energy expenditure. A high snack frequency (unhealthy crisps, sweets, and carbonated beverages), skipping daily breakfast, and emotional eating are harmful dietary habits significantly associated with a higher BMI that were found in the Saudi population (6). In addition, some Saudis are physically inactive because of the weather, culture, and lack of time and equipment (7). Sedentary lifestyles in the Saudi population as a result of sedentary jobs, long car trips, and watching screens (computer, mobile, television and games) for more than 8 hours per day contribute to the physical inactivity of Saudi adults (7).

Schoolteachers have specific environmental and psychological challenges that require ideal health conditions to meet their job requirements (8). Studies have reported that schoolteachers might not have a healthy dietary intake due to skipping breakfast, eating inadequate breakfast, and consuming foods high in fat and sugar or calorically dense snacks, which is not supported by dietary recommendations (9–11). This dietary behaviour among schoolteachers is more likely to lead to concentration problems and increase the risk of obesity (9). The change in socio-cultural and lifestyle for the Saudi population increase adults' consumption of unhealthy fast foods. The majority of schoolteachers in Saudi Arabia skip breakfast at home, especially females (74%); further, over 76% and 85% of males and females eat breakfast and/or snacks at school (5). A US study (12) found that the most frequently consumed foods by schoolteachers was candy (70%) and 33% of them provided it as a reward for students weekly. However, the consumption of cookies, doughnuts, sweetened drinks, and pizza was at 25%.

Additionally, most schoolteachers are moderately physically active (13–15), irrespective of their role in the classroom, and schoolteachers during teaching (14, 16). Among individuals, including schoolteachers, the obesity risk increases with the degree of physical inactivity, sedentary life, and dietary behaviour (17). The prevalence of obesity among schoolteachers was 30% in a Brazilian study (18) and 40% in a Saudi study in Jeddah (19). Obesity is associated with lifestyle and comorbidities among public school teachers (20). The higher obesity prevalence among schoolteachers may be related to high levels of sedentary behaviour during leisure (20) and the association of prolonged screen time, sedentary behaviour, and unhealthy eating habits (15). In addition, sitting time and watching screen devices are associated with consuming products of high energy density and low nutritional content. Globally, one in four adults is physically inactive (21), compared to one in two in Saudi Arabia (22) and one-third in South Africa (23). A Brazilian study (24) found that more than 50% schoolteachers are physically inactive, and this is as result of low levels of physical activity, high levels of TV viewing and low energy expenditure. Generally, schoolteachers sit in an orthostatic position at school 95% of the time. Furthermore, previous scientific evidence indicated health impact of increased percentages of obesity among different populations including increase rates of associated chronic diseases such as cardiovascular disease, diabetes, hypertension (25), and cancer (26). Also, studies showed other undesirable health impact for obesity including the presence of psychological disorders (27, 28), body dissatisfaction (29). Moreover, the health of schoolteachers affects teaching quality. This may indirectly affect student achievement and students' subsequent success in society due to the role of a teacher in students' lives, especially at this critical age (30). Previous literature illustrated that schoolteachers have been affected by chronic diseases globally. For example, the rates of hypertension were reported by 25%, 29% and 84% among Saudi, Indian, and Nigerian schoolteachers, respectively (31–33).

As schoolteachers are considered role models for students, the dietary intake and physical activity of schoolteachers may influence their students' behaviours. Also, teachers spend ~7 h a day, 5 days per week at schools, working with students, which allows students to copy teachers' food choices during breakfast and snack times. Therefore, schoolteachers should be positive role models for students by setting an example of healthy dietary intake and healthy lifestyle habits (34, 35). Dietary intake, physical activity levels, and obesity are important indicators of health status among schoolteachers, which may affect their work performance. Hence, the current study hypothesises that public schoolteachers who are physically active and have a normal BMI have a healthy dietary intake. To the best of our knowledge, the association between physical activity, obesity status, and dietary intake has not been examined among schoolteachers in Saudi Arabia. Additionally, previous studies regarding school nutrition and health interventions focused considerably on students, not teachers. Therefore, this study aimed to fill this gap and assess the dietary intake of certain food groups in a representative sample of public-school teachers living in Jeddah city. We further examined the association of dietary intake with physical activity and obesity among schoolteachers. The finding of this study will enhance future health interventions in schools and lead policymakers in Saudi Arabia and beyond.

## 2. Methods

### 2.1. Study design and participants

This cross-sectional study was conducted between October 2021 and March 2022 on teachers working in public schools in Jeddah, Saudi Arabia. This study was first approved by the Biomedical Ethics Research Committee of King Abdulaziz University (Reference No. 159-21). In order to collect data from schoolteachers in Jeddah city, approvals were also sought from the Education Department of Jeddah.

The inclusion criteria were male and female teachers, aged between 18 and 65 years old, working in public primary, intermediate, and high schools in Jeddah city. This study excluded teachers working in private schools. There are six administrative educational offices for public schools in Jeddah: North, East, Centre, Naseem, South, and Safa. According to the Ministry of Education statistics, the total number of male and female teachers working at public schools in Jeddah was as follows: in primary, intermediate, and high schools there were 6,046, 3,777, and 2,189 male teachers and 7,304, 4,323, and 3,219 female teachers, respectively (36). Hence, according to the online sample size calculator (37), the required sample size for the current study was 638 schoolteachers working in public schools in Jeddah.

### 2.2. Measures

An online self-reported survey was distributed electronically *via* par code and WhatsApp, e-mail to several educational offices and, in return, to all public schools in their area during the school year. A closed-ended multiple-choice questionnaire was used to collect and assess data in four categories: sociodemographic characteristics, health characteristics, health behaviours, and dietary intake. The online self-reported survey was used because it allowed the collection of data from a large and representative sample in a short time. At the beginning of the online survey, schoolteachers were informed about the research objectives and procedures, and their anonymity and voluntary participation in this research were guaranteed. Since the survey was distributed online, consent was obtained by adding a consent statement, respondents provided consent through this to participate in the research. Thus, only those who agreed to participate were able to complete the survey.

#### 2.2.1. Sociodemographic characteristics

Sociodemographic characteristics of schoolteachers included age (18–65), sex (male; female), marital status (single, married, divorced, widowed), education level (diploma; bachelor; postgraduate), school stage (primary; secondary; high), and years of teaching experience (1–5; 6–10; 11–15;16–20; and above 20 years).

#### 2.2.2. Health characteristics

Self-reported health characteristics, including the presence of chronic disorders (e.g., diabetes, hypertension, coronary heart disease, high cholesterol levels) and self-reported height and weight, were collected to determine body mass index (BMI). BMI in kg/m^2^ was categorised as normal weight (BMI between 18.5 and 24.99), overweight (BMI ≥ 25.0), or obese (BMI ≥ 30.0). BMI was classified according to the cut-off points of the World Health Organization (WHO) (38).

#### 2.2.3. Health behaviours

Questions were related to participants' health behaviours, including physical activity, cigarette smoking, and dietary intake. For physical activity, questions were adapted from previous study conducted in Saudi Arabia (39). Participants were asked to self-report their physical activity, such as walking, running, or swimming, with the responses being classified as never, rarely, 1–2 per week, 3–4 per week, and more than 5 per week. In the analysis, we categorised them into two groups: low (participants who exercised two times per week or less) and high (participants who exercised more than three times per week) physical activity (39). Smoking was assessed by asking the participants about their cigarette smoking habits during the previous year. The responses were categorised as smoker, ex-smoker, and non-smoker.

#### 2.2.4. Dietary intake

A short, non-quantitative, validated Food Frequency Questionnaire (FFQ) was used to assess the weekly frequency (days/week) of dietary intake for 14 food items, including fruits, fruit juices, salads, vegetables, dairy products, beans and legumes, cereals, wholegrain bread, snacks, sweets, soft drinks, white and red meat, and fish. The validated questionnaire was adopted from Cleghorn et al. (40). Each FFQ item had seven frequency options including (never, once a week, 2 to 4 times per week, 5 to 6 times per week, 1 to 2 times per day, 3 to 4 time per day, or 5 or more times per day). The participants were asked on average how often they consumed these 14 food items during the past 12 months. Based on participants responses, consumption was categorised as “low intake” or “high intake”. A consumption frequency of at least 5 days/week was defined as high intake, whereas low intake was defined as <5 days/week. These categories were based on a study done by Delfino et al. (15).

### 2.3. Statistical analysis

All of the data presented in the current study were categorical variables. Descriptive data of the study participants are shown in tables as frequencies (*n)* and percentages (%). Correlations between categorical variables (demographic characteristics, gender, and dietary intake) were examined using the chi-square test. Logistic regression analyses were performed to obtain odds ratios (ORs) and 95% CIs of dietary intake by physical activity levels and BMI status. All models were adjusted for a selected set of covariates, including sex, age, marital status, education level, years of experience, health status, and smoking status. In the analysis model, physical activity and BMI status were considered the explanatory variables, while the dietary intake was considered the outcome variables. For all analyses, the *P* < 0.05 was considered borderline significant. However, due to multiple testing of 14 food groups, we applied Bonferroni correction, and the statistical significance level was set at *P* = 0.003. Statistical analyses were performed using SPSS software version 28.0.

## 3. Results

The demographic characteristics of public-school teachers from Jeddah, Saudi Arabia, based on gender are shown in Table 1. The sample consisted of 640 schoolteachers; 72.3% were female, and 27.7% were male. Among the study participants, 74.1% were married, and 68.2% were aged between 35 and 54 years old. The majority of the study participants (83.6%) held a bachelor's degree. There were significant differences between male and female teacher in marital status and education level. In comparison to men, the percentage of widowed, single, and divorced women teachers was higher than that of men (*P* = 0.03). A higher percentage of women teachers held diplomas, while a higher percentage of men held postgraduate degree (*P* < 0.001). Most of the participants (40.9%) taught in high schools, and 36.6% had more than 20 years of teaching experience. Approximately 36.6% of the schoolteachers included in this study were healthy, 13.4% were diagnosed with diabetes, and 10.2% were diagnosed with hypertension. The mean BMI was 27.2 kg/m^2^ (SD = 4.9), and 34.4% of the participants were in the normal weight range, whereas 40.8% and 24.5% of the participants were overweight and obese, respectively. There were more overweight men teachers than women teachers, while there were more obese women teachers than men teachers (*P* = 0.003). In general, the percentage of non-smokers was 83.8%. There were more non-smoker women teachers (94%) than non-smoker men teachers (57.1%) (*P* < 0.001). A total of 53.6% of the participants were classified as highly physically active. Men teachers were more active than women teachers (*P* = 0.03). Table 2 presented dietary intake according to gender. There was no significant difference in dietary intake between men teachers and women teachers except that men teachers consume more soft drinks than women teachers (*P* = 0.002).

**Table 1:** Baseline characterisation of schoolteachers from public school stratified by gender.

**Characterisation**	**Total (*n* = 640)**	**Male (*n* = 177)**	**Female (*n* = 463)**	***P*-value**
**Age**				
18–24	58 (9.1)	7 (4.0)	51 (11.0)	0.07
25–34	71 (11.1)	18 (10.2)	53 (11.4)	
35–44	194 (30.3)	59 (33.3)	135 (29.2)	
45–54	243 (38.0)	70 (39.5)	173 (37.4)	
55–65	74 (11.6)	23 (13.0)	51 (11.0)	
**Marital status**				
Single	106 (16.6)	24 (13.6)	82 (17.7)	0.03
Married	474 (74.1)	142 (80.2)	332 (71.7)	
Divorced	44 (6.9)	11 (6.2)	33 (7.1)	
Widow	16 (2.5)	0 (0)	16 (3.5)	
**Education level**				
Diploma	66 (10.3)	11 (6.2)	55 (11.9)	<0.001
Bachelor	535 (83.6)	144 (81.4)	391 (84.4)	
Postgraduate	39 (6.1)	22 (12.4)	17 (3.7)	
**School stage**				
Primary	221 (34.5)	65 (36.7)	156 (33.7)	0.61
Secondary	157 (24.5)	45 (25.4)	112 (24.2)	
High	262 (40.9)	67 (37.9)	195 (42.1)	
**Experience years**				
1–5	101 (15.8)	29 (16.4)	72 (15.6)	0.12
6–10	116 (18.1)	21 (11.9)	95 (20.5)	
11–15	99 (15.5)	33 (18.6)	66 (14.3)	
16–20	90 (14.1)	27 (15.3)	63 (13.6)	
Above 20	234 (36.6)	67 (37.9)	167 (36.1)	
**Health condition**				
Healthy	417 (65.1)	109 (61.6)	308 (66.5)	0.32
Having diabetes	86 (13.4)	26 (14.7)	60 (13.0)	
Having heart disease	12 (1.9)	6 (3.4)	6 (1.3)	
Having hypertension	65 (10.2)	21 (11.9)	44 (9.5)	
High cholesterol	60 (9.4)	15 (8.5)	45 (9.7)	
**Smoking cigarette**				
Smoker	65 (10.2)	47 (26.6)	18 (3.9)	<0.001
Quit smoking	39 (16.4)	29 (16.4)	10 (2.2)	
Non-smoker	536 (83.8)	101 (57.1)	435 (94.0)	
**Physical activity**				
Low active	297 (46.4)	70 (39.5)	227 (49.0)	0.03
High active	343 (53.6)	107 (60.5)	236 (51.0)	
**BMI status**				
Normal weight	220 (34.3)	53 (29.9)	167 (36.6)	0.003
Overweight	258 (40.3)	91 (51.4)	167 (36.6)	
Obese	155 (24.2)	33 (18.6)	122 (26.8)	

Data presented as frequency (%).

*P*-value was calculated based on chi-square.

**Table 2:** Dietary intake based on gender of public schoolteachers.

	**Total**	**Male**	**Female**	***P*-value**
**Fruit**				
Low intake	525 (82)	142 (80.2%)	383 (82.7%)	0.49
High intake	115 (18)	35 (19.8%)	80 (17.3%)	
**Fruit juice**				
Low intake	559 (87.3)	154 (87.0%)	405 (87.5%)	0.89
High intake	81 (12.7)	23 (13.0%)	58 (12.5%)	
**Salad**				
Low intake	460 (71.9)	127 (71.8%)	333 (71.9%)	1.00
High intake	180 (28.1)	50 (28.2%)	130 (28.1%)	
**Vegetables**				
Low intake	500 (78.1)	142 (80.2%)	358 (77.3%)	0.45
High intake	140 (21.9)	35 (19.8%)	105 (22.7%)	
**Bean and legumes**				
Low intake	564 (88.1)	150 (84.7%)	414 (89.4%)	0.10
High intake	76 (11.9)	27 (15.3%)	49 (10.6%)	
**Cereal**				
Low intake	567 (88.6)	161 (91.0%)	406 (87.7%)	0.26
High intake	73 (11.4)	16 (9.0%)	57 (12.3%)	
**Whole grain bread**				
Low intake	462 (72.2)	125 (70.6%)	337 (72.8%)	0.62
High intake	178 (27.8)	52 (29.4%)	126 (27.2%)	
**Dairy**				
Low intake	416 (65)	107 (60.5%)	309 (66.7%)	0.13
High intake	224 (35)	70 (39.5%)	154 (33.3%)	
**Snacks**				
Low intake	560 (87.5)	154 (87.0%)	406 (87.7%)	0.79
High intake	80 (12.5)	23 (13.0%)	57 (12.3%)	
**Sweet**				
Low intake	500 (78.1)	144 (81.4%)	356 (76.9%)	0.24
High intake	140 (21.9)	33 (18.6%)	107 (23.1%)	
**Soft drinks**				
Low intake	560 (87.5)	143 (80.8%)	417 (90.1%)	0.002
High intake	80 (12.5)	34 (19.2%)	46 (9.9%)	
**Red meat**				
Low intake	559 (87.3)	150 (84.7%)	409 (88.3%)	0.23
High intake	81 (12.7)	27 (15.3%)	54 (11.7%)	
**White meat**				
Low intake	450 (70.3)	117 (66.1%)	333 (71.9%)	0.17
High intake	190 (29.7)	60 (33.9%)	130 (28.1%)	
**Fish**				
Low intake	599 (93.6)	158 (89.3%)	400 (86.4%)	0.35
High intake	41 (6.4)	19 (10.7%)	63 (13.6%)	

Data presented as frequency (%).

*P*-value was calculated based on chi-square.

The association between physical activity levels and dietary intake is shown in Table 3. Schoolteachers with low levels of physical activity exhibited a decreased consumption of salad (OR = 0.6, 95% CI 0.4–0.9; *P* = 0.02), vegetables (OR = 0.6, 95% CI 0.3–0.9; *P* = 0.01), beans and legumes (OR = 0.4, 95% CI 0.2–0.7; *P* = 0.005), wholegrain bread (OR = 0.6, 95% CI 0.4–0.9; *P* = 0.03), dairy products (OR = 0.6, 95% CI 0.4–0.9; *P* = 0.01), snacks (OR = 0.5, 95% CI 0.2–0.8; *P* = 0.01), and fish (OR = 0.4, 95% CI 0.2–0.9; *P* = 0.04) compared to those with high levels of physical activity. However, the difference was borderline significant. Only fruit intake was considered statistically significant (OR = 0.4, 95% CI 0.3–0.7; *P* = 0.003). The associations between BMI and dietary intake are shown in Table 4. Obese schoolteachers had a decreased consumption of fruits (OR = 0.3, 95% CI 0.2–0.7; *P* = 0.007) and white meat (OR = 0.5, 95% CI 0.3–0.9; *P* = 0.03) than schoolteachers in the normal weight group. Among the enrolled schoolteachers, the risk of being obese was associated with a decreased intake of vegetables (OR = 0.5, 95% CI 0.3–0.9; *P* = 0.05) compared to the group with normal body weight, but this did not reach statistical significance. However, the difference was borderline significant, and none of these associations were statistically significant.

**Table 3:** Odds ratios and 95% CIs of food group intake according to physical activity levels of public schoolteachers.

	**Odds**	**95% CI**	***P*-value**
**Fruit**			
Low PA	0.4	0.3–0.7	0.003
High PA	1	Reference	
**Fruit juice**			
Low PA	0.8	0.4–1.4	0.50
High PA	1	Reference	
**Salad**			
Low PA	0.6	0.4–0.9	0.02
High PA	1	Reference	
**Vegetables**			
Low PA	0.6	0.3–0.9	0.01
High PA	1	Reference	
**Bean and legume**			
Low PA	0.4	0.2- 0.7	0.005
High PA	1	Reference	
**Cereal**			
Low PA	0.6	0.3–1.1	0.16
High PA	1	Reference	
**Whole grain bread**			
Low PA	0.6	0.4–0.9	0.03
High PA	1	Reference	
**Dairy**			
Low PA	0.6	0.4–0.9	0.01
High PA	1	Reference	
**Snacks**			
Low PA	0.5	0.2–0.8	0.01
High PA	1	Reference	
**Sweet**			
Low PA	0.8	0.5–1.2	0.39
High PA	1	Reference	
**Soft drinks**			
Low PA	1.1	0.7–2.1	0.51
High PA	1	Reference	
**Red meat**			
Low PA	0.5	0.3–1.0	0.05
High PA	1	Reference	
**White meat**			
Low PA	0.7	0.5–1.1	0.09
High PA	1	Reference	
**Fish**			
Low PA	0.4	0.2–0.9	0.04
High PA	1	Reference	

*P*-value was calculated using logistic regression. The model adjusted for gender, age, marital status, education level, experience years, body mass index, health status, and smoking.

PA, physical activity.

Physical activity was explanatory variables and dietary intake was outcomes variables.

**Table 4:** Odds ratios and 95% CIs of food group intake according to body mass index categories of public schoolteachers.

	**Odds**	**95% CI**	***P*-value**
**Fruit**			
Normal weight	1	Reference	
Overweight	0.6	0.4–1.1	0.13
Obese	0.3	0.2–0.7	0.007
**Fruit juice**			
Normal weight	1	Reference	
Overweight	0.7	0.4–1.4	0.41
Obese	0.6	0.2–1.3	0.21
**Salad**			
Normal weight	1	Reference	
Overweight	0.8	0.5–1.2	0.43
Obese	0.8	0.4–1.4	0.48
**Vegetables**			
Normal weight	1	Reference	
Overweight	0.7	0.4–1.2	0.28
Obese	0.5	0.3–0.9	0.05
**Bean and legume**			
Normal weight	1	Reference	
Overweight	0.6	0.3–1.2	0.22
Obese	1.5	0.7–3.2	0.21
**Cereal**			
Normal weight	1	Reference	
Overweight	0.6	0.3–1.3	0.26
Obese	1.1	0.5–2.2	0.78
**Whole grain bread**			
Normal weight	1	Reference	
Overweight	0.7	0.4–1.1	0.21
Obese	1.1	0.6–1.9	0.62
**Dairy**			
Normal weight	1	Reference	
Overweight	0.7	0.4–1.1	0.09
Obese	0.6	0.4–1.1	0.11
**Snacks**			
Normal weight	1	Reference	
Overweight	0.5	0.3–1.1	0.02
Obese	0.9	0.4–1.9	0.96
**Sweet**			
Normal weight	1	Reference	
Overweight	0.7	0.4–1.2	0.29
Obese	1.1	0.6–1.9	0.69
**Soft drinks**			
Normal weight	1	Reference	
Overweight	1.3	0.7–2.4	0.38
Obese	1.3	0.6–2.8	0.39
**Red meat**			
Normal weight	1	Reference	
Overweight	1.2	0.6–2.1	0.50
Obese	0.6	0.3–1.4	0.32
**White meat**			
Normal weight	1	Reference	
Overweight	0.8	0.5–1.2	0.40
Obese	0.5	0.3–0.9	0.03
**Fish**			
Normal weight	1	Reference	
Overweight	0.8	0.3–1.9	0.68
Obese	1.5	0.6–3.7	0.37

*P*-value was calculated using logistic regression. The model adjusted for gender, age, marital status, education level, experience years, physical activity, health status, and smoking.

BMI status was explanatory variables and dietary intake was outcomes variables.

## 4. Discussion

To prevent and control the rise of obesity prevalence and its complications, it is fundamental to establish healthy eating habits in the early development of a child (41). Consequently, the most effective way to promote health in the education system is through schoolteachers who can encourage healthy eating (41). Studies show that being proactive can improve health outcomes and promote healthy lifestyles (42, 43). In addition, the essential aspects of implementing health promotion in schools have been achieved with the aid of teachers' interest and engagement (43, 44). Thus, this study aimed assess the dietary intake of certain food groups in a representative sample of public-school teachers living in Jeddah city. Also, it aimed to examine the association of dietary intake with physical activity and obesity among schoolteachers. The main study findings regarding health behaviours of Saudi public-school teachers showed that the prevalence of overweight and obesity was 65%, the percentage of non-smokers was 84%, and 54% of the study participants met the definition of high physical activity. Our data show that physically active teachers consume more fruits, vegetables, beans, legumes, and dairy products than teachers who are less physically active. Less red meat and fewer snacks were consumed by teachers who were less physically active than by those who were more active. Compared to teachers with normal weight, those with a high BMI consumed fewer fruits, vegetables, snacks, and white meat. Hence, the main hypothesis was confirmed. To the best of our knowledge, no previous studies have examined the association of dietary intake with physical activity and obesity among schoolteachers in Saudi Arabia.

In this study, the obesity prevalence of schoolteachers was twice as high as the global obesity prevalence of adults (24.5 vs. 13.0%), and further analysis of the determinants is needed to investigate this alarming finding. This increased obesity frequency is consistent with earlier findings by Lizana et al. (45) reporting an obesity frequency of 25.7% among 70 rural Chilean teachers. Other studies among different populations, including 305 Tanzanian health workers, teachers, and bankers reported an even higher obesity prevalence (37.8%) with the prevalence of overweight and obesity among teachers reported by 62.6% in Tanzanian (46), which was similar to the results of the current study (65% when considering both overweight and obesity). It should be emphasised that the age of the current study population may have contributed to the increased obesity prevalence, as obesity increases progressively with age, reaching its peak in age groups from 40 to 60 years (47); these age groups were predominant in our study population. The risk of developing a several non-communicable diseases, including obesity, cardiovascular diseases, diabetes and hypertension increases with age (48). The results of the current study also revealed that the number of overweight men teachers was higher than that of women teachers, while that of obese women teachers was higher than that of men teachers (*P* = 0.003). However, the reason for such a difference may be the hormonal differences between men and women, genetic factors, and variations in clinical severity are also thought to play a role in the interaction influence of sex in the correlations with obesity (48). Previous studies conducted in Saudi Arabia reported similar results as men had higher levels of overweight and women had higher levels of obesity (49).

The present study demonstrated that the level of physical activity in Saudi Arabia has increased, although it is still relatively low. According to the Saudi STEPwise survey, the prevalence of moderate and high levels of physical activity among Saudis aged 15–64 years was 32.3% (50). The level of physical activity reported in this study was 54%. Despite not being comparable to our study in methodology or national representativeness, other studies among Saudi adults aged 15 and older have reported increases in physical activity, ranging from 50 to 70%.(7, 51). According to the WHO, around 30% of the world's population and 30–70% of those living in countries in the eastern Mediterranean region do not meet the recommended minimum level of physical activity (52). In the present study, women teachers were found to be less physically active than men teachers. A possible explanation for this might be that men in Saudi Arabia generally have more opportunities than women to engage in outdoor physical activity especially considering the hot weather in the kingdom, which may affect women who wear hijab to exercise (49). However, there are plenty of health clubs for both sexes; nevertheless, the high cost for health clubs might be an issue that impact practising physical activity (49). It is notable that women in the Middle East have generally been reported to be less physically active than men (53, 54), and lower than that in many developed countries (53).

The current study findings indicated that the consumption of food from various categories was considered poor, as only a small percentage of schoolteachers' food intake met the dietary recommendations. These results were consistent with the findings of a nationally representative survey on the Saudi adult population that revealed that the consumption of fruits, vegetables, beans, legumes, dairy products, and fish did not meet the dietary recommendations (55). Unhealthy eating habits are the leading risk factor for poor health, with an estimated 11.3 million attributed deaths and 241.4 million attributed disability-adjusted life years per annum globally (56). In line with our findings, the Global Burden of Diseases, Injuries, and Risk Factors study reported that in Saudi Arabia, the average intake of fruits, vegetables, nuts, whole grains, and seafood (polyunsaturated and omega 3) fatty acids was considerably below optimal levels, whereas the consumption of red meat, processed meats, saturated fatty acids, sugar-sweetened beverages, and salt was significantly above optimal levels (57). Interventions to enhance teacher eating habits are necessary because they may positively impact both teachers' and students' health-related behaviours. This is because healthier diets are strongly associated with a lower risk of developing chronic diseases (58). This study also reported that the teachers' dietary intake did not differ by gender except that men teachers consume more soft drinks than women teachers (*P* = 0.002). Previous studies showed similar results revealed similar findings (59, 60). A study that examined the consumption pattern of sugar-sweetened beverages among college students in Jordan and its impact on their body weight, reported that men consumed greater amounts of sugar-sweetened beverages than women (*P* = 0.016) (59). This might be because women are more aware of the risk factors of soft drinks and are more concerned with their body image than men (61, 62). Another reason for such a difference reported by Xi et al. is that men drink less water than women, which leads to increased sugar-sweetened drink consumption (63). Men are also likely to consume more soft drinks overall since their overall food consumption is larger than that of women (62).

The present study findings showed that schoolteachers with high physical activity consumed more fruits, vegetables, beans, legumes, and dairy products than those with low physical activity. A sufficient degree of physical activity leads to improve health, including decreased overweight and obesity levels. Conversely, a lack of physical exercise is strongly associated with an elevated risk of cardiometabolic and vascular diseases (64). This is probably because most study participants had university degrees (bachelor's and postgraduate), which indicates that people who are well-educated are more aware about nutritional aspects and have a favourable attitude towards leading a healthy lifestyle (54). Previous studies have shown a close relationship between exercise and healthy food choices (65–67), which appears to be associated with improved health knowledge and awareness, as well as perceiving a healthy diet and physical activity requirements as beneficial and practical (68). Similarly, a study concluded that low levels of physical activity were associated with inadequate fruit and vegetable intake among young adults in Brazil (low consumption of fruits and vegetables by 79 and 90% respectively) (69). Additionally, in previous study among college students in Saudi Arabia physically active students tend to consume higher amounts of fruits, vegetables, and dairy products than inactive students (70). Other studies found that adults with an active lifestyle eat healthier diets including more fibre and fruits and vegetables, less saturated fat, and fewer servings of fried food and sweets than sedentary adults (65, 66, 68). Furthermore, in the current study, schoolteachers with low physical activity consumed fewer snacks and less red meat than those with high physical activity. These findings are consistent with a previous study conducted among Polish adults (71). The Polish study reported that adults who considered themselves physically active consumed less red meat and meat products (71). Although, few studies investigated the relationship between physical activity and the consumption of red meat, a previous study in adult women aged 20–50 years old reported that lower physical activity is associated with low consumption of red meat, which is consistent with our findings (72). However, in a study conducted among female undergraduate students, the high consumption of unhealthy snacks was significantly associated with increased levels of physical activity (73), which is the opposite of our findings as the present study showed that those who were physically active had higher intake of healthy food and less intake of unhealthy snacks.

The present study results showed that schoolteachers with high BMI have a decreased consumption of fruits compared to those with normal weight, which is aligned with previous studies (74). These findings are supported by the fact that a higher intake of fruits and vegetables lowers the risk of developing obesity (75). Increasing fruit and vegetable intake may also contribute to weight management because fruits and vegetables are low in energy but high in fibre and water, thereby producing a satiating effect (76). The satiating properties of fruits and vegetables lead to a reduction in energy-dense, nutrient-poor food consumption, thereby reducing overall caloric intake (76, 77). The intake of snacks, depending on the quantity and frequency of consumption, is unlikely to increase body weight. However, our findings showed that schoolteachers with high BMI consumed fewer snacks than schoolteachers with normal weight. Several studies have found that snacking frequency and daily calorie consumption from snacks have a negligible or even inverse relationship with the risk of becoming overweight or obese (78, 79). However, these associations may be confounded due to the underreporting of snacking behaviours among obese people compared to normal-weight respondents (78, 80). The consumption patterns of main meals and the choice of foods and beverages consumed as snacks appear to influence the associations between snacking, diet quality, and body weight (81). In particular, frequent snacks as substitutes for main meals may have a negligible impact on body weight and daily energy balance. Moreover, our findings indicated that less intake of vegetables is associated with increased risk of obesity. Data from the Atlantic Partnership for Tomorrow's Health Study demonstrated that increased fruit and vegetable consumption is inversely associated with BMI risk (82). Previous study showed that low vegetable intake was strongly associated with obesity risk among Japanese patients with type 2 diabetes in both sexes (83). These findings are supported by the results of a systematic review confirming that increased vegetable intake is associated with weight loss of 0.09–0.1 kg over 4 years, decreased risk of weight gain, and lower risk of overweight or obesity (84). Vegetables may contribute to the beneficial effects of weight changes, as they abundantly contain vitamins, minerals, fibres, and proteins (16, 85). In the present study findings, white meat consumption had a negative association with increased BMI. Red meat consumption has been linked to general and abdominal obesity in several epidemiological studies, but the results have been inconsistent (86, 87). Several studies have indicated that red meat consumption is positively associated with obesity (88, 89), whereas others have not found a significant correlation (90, 91). Because red meat and its products are a rich source of protein, cholesterol, and saturated fatty acids, they are considered high-energy-density foods (88). Moreover, the association between white meat consumption and obesity has received less attention in previous studies than red meat consumption, and the results in this regard have been more controversial (92, 93). According to Vergnaud et al., who assessed the association between the consumption of total meat, red meat, poultry, and processed meat in a large European population, higher poultry consumption was found to be associated with a lower risk of obesity (93), whereas Maskarinec et al. found a significant positive relationship between white meat consumption and obesity in Hawaii (94). Furthermore, a longitudinal study of ~90,000 adults whose dietary habits and anthropometric data were measured for 6.5 years showed a statistically significant relationship between the consumption of animal proteins and long-term weight gain (95).

### 4.1. Strengths and limitations

The main strength of this research is that it is the first study to examine the associations of dietary intake with physical activity and obesity using a representative sample of public school teachers living in Jeddah. In our analysis, we adjusted for socioeconomic factors that may significantly influence dietary intake. However, the current study has certain limitations. First, information about dietary intake, physical activity level, health status and body height and weight were collected using a self-reported questionnaire, which may raise concerns about recall bias, as some participants responded in socially desirable ways for some variables, including dietary intake. Second, the dietary intake was not measured using the semi-quantitative FFQ; only the intake frequency, i.e., the number of days per week, was recorded. Future studies should consider FFQ which captures more food items than the one used in this study. Likewise, future studies should also consider using the semi-quantitative FFQ with both frequency and portion sizes. Another point is that body weight can affect the quantity and quality of food consumed, and vice versa (96); thus, the association between dietary intake and obesity can be ambiguous. Third, the cross-sectional design prevents drawing conclusions about causal relationships. Finally, the study findings cannot be generalised to all schoolteachers in Saudi Arabia, as this study examined only schoolteachers in Jeddah city; thus, extending the findings to other populations is not possible. Future studies should collect data from all regions in Saudi Arabia in order to generalise the findings for all the Kingdom.

## 5. Conclusions

The current study is one of the first to examine the associations of dietary intake with physical activity and obesity among schoolteachers in Saudi Arabia. Schoolteachers are a vital group that is neglected in scientific research, and they play an important role in promoting health in education systems by encouraging healthy lifestyles and healthy eating habits among students. The findings of this study revealed that more than half of the schoolteachers reported high physical activity levels; however, a high prevalence of overweight and obesity was also reported by the study participants. These high rates of overweight and obesity might increase the current or future risk of non-communicable diseases among schoolteachers. Our results also showed that high physical activity was associated with higher consumption of healthy food, including fruits, vegetables, beans, legumes, and dairy products. Conversely, higher BMI was associated with lower consumption of fruits, vegetables, snacks, and white meat. Adjustments to the lifestyle of teachers are required, aiming to increase their healthy diet and reduce several risk factors leading to cardiovascular disease, dyslipidaemia and diabetes. Schoolteachers are expected to be role models for students and future generations. Healthy schoolteachers influence the health and educational performance of students. However, health promotion interventions at schools focus on students, not teachers. Strategies to reduce the obesity risk among schoolteachers should focus on increasing the intake of a healthy diet, as well as increasing physical activity. Nutrition education programs are vital to encourage schoolteachers in Saudi Arabia to consume healthy food and adopt a healthy lifestyle, thereby enhancing public health.

## Data availability statement

The original contributions presented in the study are included in the article/supplementary material, further inquiries can be directed to the corresponding author.

## Ethics statement

The studies involving human participants were reviewed and approved by Education Department of Jeddah City and the Biomedical Ethics Research Committee of King Abdulaziz University (Reference No. 159-21). The patients/participants provided their written informed consent to participate in this study.

## Author contributions

Conceptualisation: NAlm, IS, and NAlj. Methodology: NAlj and NAlm. Formal analysis: IS. Data collection and writing—original draft preparation: NAlm, IS, MA, MH, HW, and NAlj. All authors have read and agreed to the published version of the manuscript.
